# The temporal decline of idealism in two cohorts of medical students at one institution

**DOI:** 10.1186/1472-6920-14-58

**Published:** 2014-03-24

**Authors:** Emily M Mader, Carrie Roseamelia, Christopher P Morley

**Affiliations:** 1 Department of Family Medicine, SUNY Upstate Medical University, 750 E. Adams Street, MIMC 200, Syracuse, NY 13066, USA; 2 Department of Public Health & Preventive Medicine, SUNY Upstate Medical University, Syracuse, NY, USA; 3 Department of Psychiatry & Behavioral Sciences, SUNY Upstate Medical University, Syracuse, NY, USA

**Keywords:** Career choice, Idealism, Students, Medical, Surveys

## Abstract

**Background:**

A number of studies have indicated that students lose idealistic motivations over the course of medical education, with some identifying the initiation of this decline as occurring as early as the second year of the traditional US curricula. This study builds on prior work testing the hypothesis that a decline in medical student idealism is detectable in the first two years of medical school.

**Methods:**

The original study sought to identify differences in survey responses between first-year (MS1) and second-year (MS2) medical students at the beginning and end of academic year 2010, on three proxies for idealism. The current study extends that work by administering the same survey items to the same student cohorts at the end of their third and fourth years (MS3 and MS4), respectively. Survey topics included questions on: (a) motivations for pursuing a medical career; (b) specialty choice; and (c) attitudes toward primary care. Principle component analysis was used to extract linear composite variables (LCVs) from responses to each group of questions. Linear regression was then used to test the effect of the six cohort/time-points on each composite variable, controlling for demographic characteristics.

**Results:**

Idealism in medicine decreased (β = -.113, p < .001) while emphasis on employment and job security increased (β = .146, p < .001) as motivators of pursuing a career in medicine at each medical school stage and time period. Students were more likely to be motivated by student debt over interest in content in specialty choice (β = .077, p = .004) across medical school stages. Negative attitudes towards primary care were most sensitive to MS group and time effects. Both negative/antagonistic views (β = .142, p < .001) and negative/sympathetic views (β = .091, p < .001) of primary care increased over each stage.

**Conclusions:**

Our results provide further evidence that declines in medical student idealism may occur as early as the second year of medical education. Additionally, as students make choices in their medical careers, such as specialty choice or consideration of primary care, the influences of job security, student debt and social status increasingly outweigh idealistic motivations.

## Background

Idealism in medicine can be defined as the pursuit of the improved quality of life and relief of suffering for all humankind, with an emphasis on the provision of medical practice that focuses on providing service-oriented, interpersonal care to underserved or disadvantaged populations, as well as a concern for the health of society as a whole [1]. Idealism toward medical practice and patient care may be viewed as a fundamental quality for medical professionals [2,3], and is often reflected in a commitment to preventive care and population health, as well as the pursuit of medical specialties such as primary care or family medicine. However, a growing body of literature generally places a decrease in student idealism around the beginning of the third year of medical school [4-7]. Other studies additionally indicate that medical student idealism may start to decline as early as the end of the first or beginning of the second year of medical school [2,8,9]. Documenting the existence of a decline in idealism as a generalizable truth, and identifying potential flexion points in the development of medical students into physicians, is crucial if one values the preservation of idealism in medicine.

As Sethia and others have argued recently, the preservation of idealism in the face of pragmatic economic realities is vital to the delivery of high quality, compassionate care [10-12], both on the level of the physician-patient dyad, as well as when the physician is placed in the position of making policy decisions. Idealism, as such, is a quality that cuts across specialty or practice-type choice. Additionally, idealism may play a role in the choices medical students make about career direction. As Enoch et al. have noted, students experiencing emotional exhaustion and burnout gravitate to career specialties with greater work-related lifestyle control and higher income, and away from more poorly compensated primary care or core specialties [13]. Students who lack exposure to clinical role models – persons who can best demonstrate compassionate physician-patient relationships – may experience a greater decline in idealism and in potentially related traits, such as empathy [14,15]. For example, Winseman et al. found that students perceived mentoring and clinical experience with doctors who demonstrate empathy as two highly important factors influencing their ability to be empathetic [16].

Furthermore, the cultural influences transmitted at organizational or structural levels, or the ‘hidden curriculum’ [17,18] within medical schools, may lead students to adopt technical, detached and non-humanistic views of patients [19,20]. Several studies suggest that the hidden curriculum includes a ‘bashing’ of primary care (e.g. Family Medicine, General Adult Internal Medicine, General Pediatrics) and other core specialties (e.g. General Surgery, Obstetrics & Gynaecology) by faculty and residents [20-22]. The influences of the hidden curriculum are not often outwardly acknowledged by the institution or faculty, but can have a strong impact on student attitudes and mentalities toward practice. Institutional strategies aimed at preserving idealism may therefore provide a partial remedy to burnout-inducing conditions, empathy loss, and a hidden curriculum that encourages students away from less lucrative or prestigious practice and specialty choices.

The purpose of this study was to examine changes in proxies for medical student idealism in two student cohorts, using multiple observations across the four years of the traditional medical curriculum at one institution. Existing research on medical student idealism has targeted specific points in the medical curriculum (i.e. pre-clinical training, clerkships and residencies) or, when following student cohorts, has utilized a small number of measurement periods [2,6,23]. The present study aims to build on the current literature by measuring changes in medical student idealism over a period that provided observations from six time points across the medical curriculum.

## Methods

This study builds on prior work comparing survey data of first-year (MS1) and second-year (MS2) medical students on three dimensions of idealism [9]. The previous study measured self-reported motivations for students to select medicine as a career, factors influencing their decision-making process regarding specialty choice, and opinions about primary care, using each as a proxy for and dimension of idealism. The survey was implemented twice in the same year, near the start and end of the 2010–2011 academic year (AY2010), to both MS1s and MS2s at a single medical school. Since the two cohorts were comparable groups, this provided observations of our construct of idealism at four time points over the first two years of medical school (MS1 near the beginning of the year; MS1 at the end of the year; MS2 at the beginning of the year, and MS2 at the end of the year). We followed up that study by administering the same survey items to collect data from the same student cohorts at the end of their third and fourth years (MS3 and MS4), respectively. The survey was initiated in AY2010 and concluded in the Spring of 2013, near the end of academic year 2012–2013 (AY2012).

### Survey procedures

The initial survey procedures implemented during AY2010 are described in detail in a prior report [9]. Briefly, MS1s and MS2s were surveyed via a paper instrument during a required clinical skills course at the beginning and end of AY2010. Response rates for those surveys exceeded 90%, given the “captive audience” nature of distribution. Student identifiers were stripped from responses to protect anonymity, and the study was reviewed by the institutional review board (IRB) at SUNY Upstate Medical University (US IRB Registration #00000391, Federal-Wide Assurance #00005967), which declared the study exempt from review.

The current survey was administered to all matriculated MS3s and MS4s at the end of AY2012 electronically via SurveyMonkey™ (http://www.surveymonkey.com), through email invitations. Non-responding students were sent up to three additional invitations. SurveyMonkey™ allows for tracking whether invitees have responded to a survey invitation while separating the identity of each respondent from their actual response, keeping the collected data anonymous. It is important to note that the removal of student identifiers at all stages of survey implementation inhibited the ability to link individual student responses across each measurement period. Survey completers were offered a $10 stipend for their response. The addition of the AY2012 survey was reviewed and granted an exemption by the SUNY Upstate IRB.

Respondents answered demographic questions on each survey, including race, ethnicity, gender, MS year, geographic location where secondary education was completed (in USA vs. Non-USA; rural–urban spectrum), age, marital status, and number of children. Additionally, respondents answered three Likert-scaled matrix questions:

• *How important are the following factors in considering your career in medicine?*

• *How important are the following factors in considering your choice for a specialty?*

• *Please indicate how much you agree or disagree with the following statements (indicative of attitudes about primary care).*

The three matrix questions and items are further described in Table 1. The specific items were ranked on 5-item Likert scales, ranging from “Not Important At All” to “Very Important” for the medicine and specialty questions, and a 6-point Likert scale ranging from “Completely Disagree” to “Completely Agree,” with “Neither Agree nor Disagree” as a central anchor, and an additional “Not sure” option, for the matrix of primary care attitudinal statements. Responses were scaled from 1 (Not Important At All/Completely Disagree) to 5 (Very Important/Completely Agree). Responses on the primary care statements marked “Not Sure” were incorporated into the neutral anchor category (coded as 3).

**Table 1:** Matrix questions on AY2012 survey

**How important are the following factors in considering your career in medicine? (Likert Scale 1–5; Not Important = 1; Very Important = 5)**	**How important are the following factors in considering your choice for a specialty? (Likert Scale 1–5; Not Important = 1; Very Important = 5)**	**Attitudes toward primary care (Likert Scale 1–5; Completely Disagree = 1; Completely Agree = 5)**
• Opportunities to make a difference in people’s lives	• Income expectations for the specialty	• I would like to become a primary care doctor in the future
• Intellectual climate	• Amount of education debt I have	• I am more interested in learning the skills required for my chosen specialty rather than a general set of clinical practice skills
• Desire to do primary care	• Ability to balance my work life with my family responsibilities	• Primary care knowledge is useful for all medical students
• Availability of jobs	• Content of the specialty	• Primary care should be a patient’s first contact with the health care system
• Job security	• Competitiveness of the specialty	• Medical interviewing is a fundamental tool for all medical students to learn
• Opportunity to help patients who are socially disadvantaged	• Options for fellowship training associated with the specialty	• Preventative care knowledge is essential for all medical students to learn
• Desire to serve my community	• Length of residency training associated with the specialty	• It is essential that medical students learn how to best communicate with patients
• High income potential	• The lifestyle of the specialty I am considering	• Primary care doctors mostly manage chronic health problems
• Job satisfaction	• Prestige of the specialty I am considering	• It is impossible to be an expert in such a wide field as primary care
• Status of physicians	• Career workshops and courses	• Primary care is not very intellectually stimulating
	• Opportunities to do research in this specialty	• Primary care doctors have a large work overload
	• Opportunities to provide care to underserved populations	• Primary care doctors are poorly valued by the rest of the medical profession
		• A primary care doctor is clinically competent to provide most of the health care an individual may require

### Analysis

We conducted similar procedures to those described previously [9], adding responses for the two additional time-points. Additionally, variables were created to identify the two distinct cohorts involved in the study. Cohort 1 consisted of the group surveyed at the beginning and end of their MS1 year in 2010/11 and followed up near the end of their MS3 year in 2013; Cohort 2 was the group surveyed at the beginning and end of MS2 in 2010/11 and followed up at the end of MS4 in 2013. The analytical procedures conducted for the present study were as follows:

1. The individual items under each of the three questions were compared across the three time points within each cohort, employing the Kruskall-Wallis test to assess significance of any differences. Differences in group means between the two cohorts at each time point were also examined through Analysis of Variance (ANOVA), in order to determine, a priori, whether a cohort variable would be necessary to include in regression models in a later analytic step.

2. Latent factors were extracted from responses to each matrix question via principal components analysis (PCA). Factors were identified as those exceeding an Eigenvalue of 1, and were constructed with varimax rotation. Each factor was named based upon the top factor loadings, using a threshold of .700 as indicative of a major component, and .400 as a minor component. The factors were saved as linear composite variables in the data set, each with a mean of 0 and a standard deviation of 1.

3. The MS survey groups were coded as an ordinal variable (1–6), and were then compared again across the linear composite variables extracted through PCA, using Analysis of Variance (ANOVA) to assess significance of any observed differences between mean factor scores across the six cohort/time-points.

4. To test results from step 3 in a multivariate controlled model, each factor was then entered into a stepwise ordinary least squares (OLS) linear regression procedure, modeling the effect of the ordinal cohort/time-point variable on each composite variable, controlling for demographic characteristics. Each predictor was entered as a dummy variable in models following the form:

**Factor = Constant + β**_**1 **_**MS + β**_**2**_** Cohort + β**_***k…I***_**Covariates**

a. Cohort with Time Point (coded as: MS1/T1 = 0, MS1/T2 = 1, MS2/T1 = 2, MS2/T2 = 3, MS3 = 4, MS4 = 5)

b. Cohort:Cohort1 = 1; Cohort 2 = 0

c. Race: White/Caucasian = 1/Non-White = 0

d. Ethnicity: Hispanic = 1/Not Hispanic = 0

e. Gender: Female = 1, Male = 0

f. Rural/Urban: students originally from rural areas were determined by using the Rural–Urban Commuting Area (RUCA) approximations based upon the zip code where the student attended secondary school [20]. Two dummy variables were created:

i. Rural = 1, Non-Rural = 0

ii. Urban = 1, Rural = 0

g. Marital Status: Married = 1/Not Married = 0

h. Number of Children

i. High School in the USA: Yes = 1, No = 0

Standard OLS assumptions were applied, and diagnostic tests for multicollinearity and autocorrelation were utilized in the analysis.

## Results

Response rates for each MS cohort/time-point ranged from 99.4% (MS1 at T1) to 53.9% (MS4). Distribution across characteristics was similar for all groups. A description of the sample is presented in further detail in Table 2.

**Table 2:** Demographics of the sample, by MS* group (n for MS group and response rate^)

	**MS1/T1**	**MS1/T2**	**MS2/T1**	**MS2/T2**	**MS3/T3**	**MS4/T3**
	**n = 159 99.4%**	**n = 145 90.6%**	**n = 140 87.5%**	**n = 147 91.9%**	**n = 100 57.1%**	**n = 89 53.9%**
**Gender**						
*Male*	96	82	75	81	44	48
*Female*	63	63	65	66	47	36
**Race**						
*White*	103	97	87	94	54	63
*Black/African American*	24	19	22	25	13	5
*Asian*	29	26	23	26	16	9
*Native American*	0	0	2	2	0	0
*Other*	3	3	6	0	5	1
**Hispanic**						
*Yes*	7	7	1	1	6	3
*No*	152	138	139	146	85	81
**Attended** High School in USA						
*Yes*	147	136	125	132	90	85
*No*	12	9	15	15	10	4
**Marital** status						
*Single*	151	137	107	119	78	58
*Married*	6	6	23	23	11	26
*Divorced*	1	1	3	3	0	0
**Rural origins**						
*Rural*	14	13	16	16	9	8
*Non-rural*	145	132	124	131	91	81

*MS1 = First-year medical students; MS2 = Second-year medical students; MS3 = Third-year medical students; MS4 = Fourth-year medical students; T1 = beginning of the academic year; T2 = end of the academic year.

^Sampling frame assumed to be 160 students for MS1 & MS2 surveys; 175 for MS3; 165 for MS4.

Table 3 illustrates trends over time for each Likert-scaled item within each cohort. There were significant decreases in Cohort 1 (surveyed at the beginning and end of MS1, and again at the end of MS3) in mean Likert responses to the question, “How important are the following factors in considering your career in medicine?”, including “Opportunities to make a difference in people’s lives” (p = .003), “Opportunity to help patients who are socially disadvantaged (p = .024), and “Desire to serve my community” (p = .033). Over the same time period, “Availability of jobs” (p < .001), “Job security” (p < .001), and “High income potential” (p < .001) all increased significantly for Cohort 1. Cohort 2 had similar declines in “Opportunity to help patients who are socially disadvantaged” (p = .020) and “Desire to serve my community” (p < .001), as well as a decline in “Intellectual climate” (p < .001). “Income expectations for the specialty” (p < .001) and “Amount of education debt I have” (p = .012) increased in importance for Cohort 1 members as they thought about specialty choice. Table 3 illustrates further differences over time within each cohort.

**Table 3:** Comparison of MS cohort responses to matrix questions about career choices

		**Cohort 1 (MS1/MS3)**				**Cohort 2 (MS2/MS4)**			
**Items**		**MS1 T1**	**MS1 T2**	**MS1 T3**	***p***	**MS2 T2**	**MS2 T2**	**MS4 T3**	***p***
**How important are the following factors in considering your career in medicine?**									
Opportunities to make a difference in people’s lives		4.89	4.88	4.71	.003**	4.83	4.83	4.66	NS
Intellectual climate		4.41	4.41	4.23	.067	4.52	4.47	4.13	<.001**
Desire to do primary care		3.04	3.03	3.04	NS	2.74	2.75	2.88	NS
Availability of jobs		3.55	3.54	4.26	<.001**	3.86	3.84	3.90	NS
Job security		3.80	3.80	4.40	<.001**	4.03	4.00	4.30	NS
Opportunity to help patients who are socially disadvantaged		4.71	4.15	3.84	.024*	3.89	3.90	3.51	.020*
Desire to serve my community		4.42	4.39	4.23	.033*	4.32	4.33	3.66	<.001**
High income potential		2.82	2.82	3.45	<.001**	3.36	3.32	3.25	NS
Job satisfaction		4.76	4.77	4.84	NS	4.75	4.77	4.78	NS
Status of physicians		2.69	2.65	2.84	NS	2.97	2.86	2.80	NS
**How important are the following factors in considering your choice for a specialty?**									
Income expectations for the specialty		2.81	2.76	3.43	<.001**	3.27	3.23	3.15	NS
Amount of education debt I have		2.81	2.83	3.30	.012*	3.04	2.99	3.09	NS
Ability to balance my work life with my family responsibilities		4.37	4.38	4.53	NS	4.62	4.60	4.44	NS
Content of the specialty		4.69	4.71	4.59	NS	4.70	4.69	4.70	NS
Competitiveness of the specialty		3.13	3.11	2.91	NS	3.07	3.06	2.74	.096
Options for fellowship training associated with the specialty		3.25	3.24	3.51	NS	3.47	3.40	3.58	NS
Length of residency training associated with the specialty		3.18	3.17	3.48	NS	3.44	3.48	2.98	.007**
The lifestyle of the specialty I am considering		4.20	4.20	4.36	NS	4.42	4.38	4.16	.066
Prestige of the specialty I am considering		2.20	2.14	2.41	NS	2.49	2.42	2.59	NS
Career workshops and courses		2.66	2.63	2.71	NS	2.53	2.47	2.36	NS
Opportunities to do research in this specialty		2.58	2.57	3.37	NS	2.42	2.38	3.10	NS
Opportunities to provide care to underserved populations		3.43	3.41	3.37	NS	3.28	3.28	3.10	NS
**Attitudes toward primary care**									
I would like to become a primary care doctor in the future		3.12	3.11	3.13	.079	2.97	3.13	2.62	.036*
I am more interested in learning the skills required for my chosen specialty rather than a general set of clinical practice skills.		2.79	2.75	2.69	.001**	2.82	2.69	3.45	.001**
Primary care knowledge is useful for all medical students.		4.75	4.75	4.70	NS	4.68	4.70	4.75	NS
Primary care should be a patient’s first contact with the health care system.		4.39	4.38	4.68	NS	4.36	4.68	4.47	.007**
Medical interviewing is a fundamental tool for all medical students to learn		4.89	4.92	4.87	NS	4.83	4.87	4.88	NS
Preventative care knowledge is essential for all medical students to learn.		4.83	4.85	4.70	NS	4.53	4.70	4.67	<.001**
It is essential that medical students learn how to best communicate with patients.		4.89	4.91	4.89	NS	4.86	4.89	4.87	NS
Primary care doctors mostly manage chronic health problems.		3.26	3.25	389	NS	3.79	3.89	3.81	<.001**
It is impossible to be an expert in such a wide field as primary care.		2.69	2.69	2.97	.069	3.01	2.98	3.31	.001**
Primary care is not very intellectually stimulating.		1.90	1.91	2.47	.057	2.22	2.47	2.58	<.001**
Primary care doctors have a large work overload.		3.63	3.63	4.15	NS	3.95	4.15	4.05	<.001**
Primary care doctors are poorly valued by the rest of the medical profession.		3.48	3.49	3.78	NS	3.76	3.78	3.77	.036*
A primary care doctor is clinically competent to provide most of the health care an individual may require.		3.96	3.94	4.36	.003**	3.99	4.36	4.36	<.001**

*Significant at the 0.05 level. **Significant at the 0.01 level. Likert Scale (1 = ’Not important at all’; 5 = ’Very Important’). Differences tested via Kruskall-Wallis test; *p* below 0.10 displayed; NS = Not Significant. Values equal to or less than p = .10 shown.

In order to determine whether the two cohorts responded differently to survey items, we compared mean responses to the 35 matrix items at each of the three administrations of the survey (i.e. MS1 at beginning of AY2010 compared with MS2 at beginning of AY2010, etc.). Out of the 105 total comparisons, there were statistically significant differences in 28 group mean responses. The three series of comparisons are shown in Table 4.

**Table 4:** Comparison of MS Groups at same time points on responses to matrix questions about career choices

**Items**	**MS1 T1**	**M2 T1**	***p***	**Ms1 T2**	**MS2 T2**	***p***	**MS3 T3**	**MS4 T3**	***p***
**How important are the following factors in considering your career in medicine?**									
Opportunities to make a difference in people’s lives	4.89	4.83	NS	4.88	4.83	NS	4.71	4.66	NS
Intellectual climate	4.41	4.52	NS	4.41	4.47	NS	4.23	4.13	NS
Desire to do primary care	3.04	2.74	.062	3.03	2.75	.074	3.04	2.88	NS
Availability of jobs	3.55	3.86	.032*	3.54	3.84	.44*	4.26	3.90	.025*
Job security	3.80	4.03	.099	3.80	4.00	NS	4.40	4.30	NS
Opportunity to help patients who are socially disadvantaged	4.71	3.89	.034*	4.15	3.90	.051*	3.84	3.51	.060
Desire to serve my community	4.42	4.32	NS	4.39	4.33	NS	4.23	3.66	<.001*
High income potential	2.82	3.36	<.001**	2.82	3.32	<.001**	3.45	3.25	NS
Job satisfaction	4.76	4.75	NS	4.77	4.77	NS	4.84	4.78	NS
Status of physicians	2.69	2.97	.088	2.65	2.86	NS	2.84	2.80	NS
**How important are the following factors in considering your choice for a specialty?**									
Income expectations for the specialty	2.81	3.27	.002**	2.76	3.23	.001**	3.43	3.15	NS
Amount of education debt I have	2.81	3.04	NS	2.83	2.99	NS	3.30	3.09	NS
Ability to balance my work life with my family responsibilities	4.37	4.62	.013*	4.38	4.60	.026*	4.53	4.44	NS
Content of the specialty	4.69	4.70	NS	4.71	4.69	NS	4.59	4.70	NS
Competitiveness of the specialty	3.13	3.07	NS	3.11	3.06	NS	2.91	2.74	NS
Options for fellowship training associated with the specialty	3.25	3.47	NS	3.24	3.40	NS	3.51	3.58	NS
Length of residency training associated with the specialty	3.18	3.44	.080	3.17	3.48	.040*	3.48	2.98	.008**
The lifestyle of the specialty I am considering	4.20	4.42	.066	4.20	4.38	NS	4.36	4.16	NS
Prestige of the specialty I am considering	2.20	2.49	.042*	2.17	2.42	.072	2.41	2.59	NS
Career workshops and courses	2.66	2.53	NS	2.63	5.47	NS	2.71	2.36	.054*
Opportunities to do research in this specialty	2.58	2.42	NS	2.57	2.38	NS	3.37	3.10	NS
Opportunities to provide care to underserved populations	3.43	3.28	NS	3.41	3.28	NS	3.37	3.10	NS
**Attitudes toward primary care**									
I would like to become a primary care doctor in the future.	3.12	2.97	NS	3.11	3.13	NS	3.13	2.62	.024*
I am more interested in learning the skills required for my chosen specialty rather than a general set	2.79	2.82	NS	2.75	2.69	NS	2.69	3.45	<.001*
Primary care knowledge is useful for all medical students.	4.75	4.68	NS	4.75	4.70	NS	4.70	4.75	NS
Primary care should be a patient’s first contact with the health care system.	4.39	4.36	NS	4.38	4.68	NS	4.68	4.47	.060
Medical interviewing is a fundamental tool for all medical students to learn.	4.89	4.83	NS	4.92	4.87	.062	4.87	4.88	NS
Preventative care knowledge is essential for all medical students to learn.	4.83	4.53	<.000**	4.85	4.70	<.001**	4.70	4.67	NS
It is essential that medical students learn how to best communicate with patients	4.89	4.86	NS	4.91	4.89	NS	4.89	4.87	NS
Primary care doctors mostly manage chronic health problems.	3.26	3.79	<.001**	3.25	3.89	<.001**	3.89	3.81	NS
It is impossible to be an expert in such a wide field as primary care	2.69	3.0	.035*	2.69	2.97	.016*	2.97	3.31	0.76
Primary care is not very intellectually stimulating	1.90	2.22	.010*	1.91	2.47	.014*	2.47	2.58	NS
Primary care doctors have a large work overload.	3.63	3.95	.006**	3.63	4.15	.005**	4.15	4.05	NS
Primary care doctors are poorly valued by the rest of the medical profession.	3.48	3.76	0.22*	3.49	3.78	.033*	3.78	3.77	NS
A primary care doctor is clinically competent to provide most of the health care an individual may	3.96	3.99	NS	3.94	4.36	NS	4.36	4.36	NS

*Significant at the 0.05 level. **Significant at the 0.01 level. Likert Scale (1 = ’Not important at all’; 5 = ’Very Important’). Differences tested via Kruskall-Wallis test; *p* below 0.10 displayed; NS = Not Significant. Values equal to or less than p = .10 shown.

PCA analysis of each set of matrix question items revealed four factors in each set. For the question concerning motivators for pursuing a career in medicine, PCA revealed factors we titled as “Idealism in medicine”, “Employment and job security”, “Status and income”, and “Career satisfaction.” Regarding specialty choice, factors included “Prestige and income”, “Lifestyle and family”, “Idealism and educational experience”, and “Debt over interest in content.” For attitudes towards primary care, a factor representing high valuation for primary care skills such as medical interviewing, communication, preventive care, and general primary care knowledge, which we entitled “Value of primary care skills”, emerged as the factor with the highest explanatory power. Additionally, a “Negative/antagonistic view of primary care” and a “Negative/sympathetic view of primary care” emerged, with the Negative/antagonistic factor including items that suggested primary care providers treat principally chronic health problems, that primary care is too broad to allow for true expertise in any area, and that described disinterest in learning primary care skills that were not relevant to a chosen specialty. The Negative/sympathetic view, on the other hand, focused upon primary care being poorly valued and overworked, relative to the rest of the medical profession. Another factor (“Considering a primary care career”) was primarily characterized by responses to the statement “I would like to become a primary care doctor in the future”, along with other statements that presented favourable views of primary care. Details of the factors, items, percentage of variance explained by each factor, and loadings for each item are described in Table 5. Additionally, Table 6 illustrates the means for linear composite variables derived from the PCA procedure for each time point and group.

**Table 5:** Linear composite variables (LCV) derived via principal component analysis, with varimax rotation

**Factor (% of variance)**	**Items (Component score) (n = 780^)**
**How important are the following factors in considering your career in medicine?**	
Idealism in medicine (21.023)	Desire to serve my community (**0.805**)
	Opportunity to help patients who are socially disadvantaged (**0.753**)
	Opportunities to make a difference in people’s lives (**0.732**)
	Desire to do primary care (0.519)
Employment and job security (20.455)	Availability of jobs (**0.897**)
	Job security (**0.883**)
Status and income (14.153)	Status of physicians (**0.881**)
	High income potential (0.676)
Career satisfaction (12.239)	Intellectual climate (**0.725**)
	Job satisfaction (0.536)
**How important are the following factors in considering your choice for a specialty?**	
Prestige and income (19.883)	Prestige of the specialty I am considering (**0.736**)
	Competitiveness of the specialty (**0.721**)
	Options for fellowship training associated with the specialty (0.645)
	Income expectations for the specialty (0.542)
	Opportunities to do research in this specialty (0.492)
Lifestyle and family (17.080)	The lifestyle of the specialty I am considering (**0.826**)
	Ability to balance my work life with my family responsibilities (**0.799**)
	Length of residency training associated with the specialty (0.569)
Idealism and educational experience (11.772)	Opportunities to provide care to underserved populations (**0.808**)
	Career workshops and courses (0.570)
Debt over interest in content (10.498)	Amount of education debt I have (0.532)
	Content of the specialty (-**0.794**)
**Attitudes toward primary care**	
Value of primary care skills (19.239)	Medical interviewing is a fundamental tool for all medical students to learn (**0.827**)
	It is essential that medical students learn how to best communicate with patients (**0.738**)
	Preventative care knowledge is essential for all medical students to learn (**0.700**)
	Primary care knowledge is useful for all medical students (0.694)
Negative/antagonistic view of primary care (13.139)	Primary care doctors mostly manage chronic health problems (**0.750**)
	It is impossible to be an expert in such a wide field as primary care (**0.750**)
	I am more interested in learning the skills required for my chosen specialty rather than a general set of clinical practice skills (0.550)
Considering primary care career (11.446)	I would like to become a primary care doctor in the future (**0.702**)
	A primary care doctor is clinically competent to provide most of the health care an individual may require (0.558)
	Primary care should be a patient’s first contact with the health care system (0.482)
	Primary care is not very intellectually stimulating (-0.558)
Negative/sympathetic view of primary care (10.675)	Primary care doctors are poorly valued by the rest of the medical profession (**0.829**)
	Primary care doctors have a large work overload (**0.761**)

^Sample included all medical student groups. Major components (≥0.700) are listed in bold.

**Table 6:** Distribution of mean composite variable scores for factors derived from PCA across MS groups and time points

**Factors**	**MS1/T1**	**MS1/T2**	**MS2/T1**	**MS2/T2**	**MS3**	**MS4**	***p***
	**(n = 159)**	**(n = 145)**	**(n = 140)**	**(n = 147)**	**(n = 100)**	**(n = 89)**	
**Idealism in medicine**	0.226	0.202	0.004	- 0.005	- 0.154	- 0.551	<0.001
**Employment and job security**	- 0.225	- 0.216	- 0.007	0.006	0.443	0.254	<0.001
**Status and income**	- 0.122	- 0.149	0.204	0.128	0.012	- 0.072	0.017
**Career satisfaction**	0.008	0.013	0.146	0.110	- 0.206	- 0.205	0.029
**Prestige and income**	- 0.047	- 0.068	0.050	- 0.005	0.057	0.065	NS
**Lifestyle and family**	- 0.126	- 0.119	0.155	0.141	0.106	- 0.160	0.019
**Idealism and educational experience**	0.115	0.100	- 0.070	- 0.087	0.092	- 0.217	0.077
**Debt over interest in content**	- 0.116	- 0.145	0.014	0.021	0.318	0.041	0.013
**Value of primary care skills**	0.060	0.117	- 0.314	- 0.117	- 0.071	0.024	NS
**Negative/antagonistic view of primary care**	- 0.287	- 0.314	0.072	0.100	0.222	0.539	<0.001
**Considering primary care career**	0.092	0.046	- 0.085	- 0.065	0.148	- 0.162	NS
**Negative/sympathetic view of primary care**	- 0.274	- 0.268	0.145	0.128	0.272	0.221	<0.001

Differences across groups measure via ANOVA, post-hoc Tukey HSD. NS = Not Significant.

Each of the 12 linear composite variables, representing each factor extracted from the PCA procedures, was entered as the dependent variable in a linear regression to examine the effect of medical school year and time point, controlling for Cohort and other demographic variables. The variable representing “Idealism in medicine” decreased at each medical school stage and time period (β = -.113, p < .001), while “Employment and job security” increased across the same MS stages and times (β = .146, p < .001). Over the same points, “Status and income” increase very slightly, though not significantly, as a factor influencing a career in medicine; “Career satisfaction” decreased a small but statistically significant amount (β = -.081, p = .002). In terms of factors influencing specialty choice, none emerged as significant, with the exception of “Debt over interest in content” (β = .077, p = .004), indicating that content interest dropped as debt mounted. Negative attitudes towards primary care were most sensitive to MS group and time effects. “Negative/antagonistic” views increased over each stage (β = .142, p < .001), as did “Negative/sympathetic” views of primary care (β = .091, p < .001). Additional details about the OLS models, including model parameters, covariate estimates, and other information are included in Table 7.

**Table 7:** Results of backward stepwise linear regression analyses of each factor, modeled^ as an outcome of MS group

**Factors (n = 780)**	**Constant**	**MS year/time**	**Cohort 1 (MS1/3)**	**Covariates**	***R***^***2 ***^ ***(F sig)***
**How important are the following factors in considering your career in medicine?**					
**Idealism in medicine**	.453	-.113 (<.001)	.020 (NS)	Hispanic (.540, p = .005); White (-.330, p < .001)	.079 (<.001)
**Employment and job security**	-.207	.146 (<.001)	.116 (NS)	# Children (-.313, p < .001); White (-.254, p < .001)	.059 (<.001)
**Status and income**	.371	.001 (NS)	-.216 (.014)	Female (-.281, p = .022); Hispanic (-.477, p = .021); White (-.165, p = .031); Married (-.237, p = .037)	.051 (<.001)
**Career satisfaction**	.157	-.081 (.002)	-.247 (.005)	Rural (-.281, p = .022); White (.269, p = .001)	.034 (<.001)
**How important are the following factors in considering your choice for a specialty?**					
**Prestige and income**	.203	.035 (NS)	.069 (NS)	Married (-.447, p < .001); White (-.189, p = .015); Female (-.148, p = .046)	.034 (<.001)
**Lifestyle and family**	-.021	-.005 (NS)	-.143 (NS)	Female (.231, p = .002)	.018 (.004)
**Idealism and educational experience**	.259	-.025 (NS)	.127 (NS)	White (-.438, p < .001); Hispanic (.546, p = .006)	.067 (<.001)
**Debt over interest in content**	.025	.077 (.004)	.089 (NS)	White (-.368, p < .001)	.042 (<.001)
**Attitudes toward primary care**					
**Value of primary care skills**	-.269	.014 (NS)	.233 (.008)	Married (.443, p < .001); Female (.190, p = .009); Number of Children (-.358, p = .018)	.036 (<.001)
**Negative/antagonistic view of primary care**	.072	.142 (<.001)	-.132 (NS)	White (-.437, p < .001); Rural (-.231, p = .05)	.125 (<.001)
**Considering primary care career**	.129	.003 (NS)	.177 (.042)	White (-.397, p < .001); Rural (.348, p = .005)	.047 (<.001)
**Negative/sympathetic view of primary care**	.167	.097 (<.001)	-.110 (NS)	Number of Children (.302, p = .031)	.049 (<.001)

^MS Group coded as: (MS1/T1 = 0, MS1/T2 = 1, MS2/T1 = 2, MS2/T2 = 3, MS3 = 4, MS4 = 5), Controlling for cohort (MS1/3 = 1, vs. MS2/4 = 0), race (White = 1), ethnicity (Hispanic = 1), rural secondary school graduation (1) origin, gender (female = 1), marital status (married = 1). Predictors displayed are those left in the final model produced by stepwise entry of covariates.

## Discussion

Our results provide further evidence that medical school students experience a decrease in measures of idealism as they progress through their education. The MS1 students, at both T1 and T2, possess the highest association with factors representing idealism in medicine and value of primary care. This association fades as responses progress through MS2, MS3 and MS4 student groups. In fact, as responses progress from the MS1/T1 to MS4 student group, the association shifts away from the factors representing idealism in medicine and value of primary care to factors reflecting job security, status and income, as well as negative views of primary care. This trend is present when looking temporally across MS student groups, as well as when viewing student groups by cohort.

Additionally, as students make choices in their medical careers, such as specialty choice or consideration of primary care, the influences of job security, student debt and social status increasingly outweigh idealistic motivations. Reasons for this shift away from idealistic motivations my stem from the increasing amount of debt students acquire as they progress through medical school [24]. The loss of idealism and value of primary care may also be partially due to a hidden curriculum that turns students away from relatively less lucrative and more service-oriented careers, such as primary care [25-27]. These influences, in addition to other external pressures on career choice such as social expectations or anticipated income, may override or replace some students’ initial idealistic motivations for a career in medicine.

It is important to note the limitations of this study. The main limitation of this research is that the data obtained for comparison originate from two cohorts and do not track individual students across time. Tracking individual students from MS1 through MS4 would provide a stronger assessment of attitudinal change; this approach was not feasible for the present study due to restrictions on the use of individual identifiers by the institutional review board. The inability to track individual students across time precluded our ability to confirm the composition of the cohorts as identical at each measurement period. However, there were no substantial changes to the curriculum, nor to the admission requirements between the two cohort groups, and the cohorts were demographically comparable. We also did not track student age, which may be related to idealism, as age would potentially have caused outlier students to be easily identifiable.

Another limitation is the fact that this study was conducted at a single institution, and follow-up at other institutions may be warranted. At present, the slightly more white and male population of this single institution, relative to the general US medical school population, may inhibit generalizability to a small extent. Additionally, the decrease in survey response rates across cohort/time-points may have introduced participation bias in the measurement of attitudinal change across medical education, as the attitudes of those students who completed each survey may not necessarily reflect the attitudes of those students who did not participate in all iterations of data collection. Finally, it is important to note that the instruments utilized in this study were constructed to track general student interests and not idealism per se*.* However, unlike other relevant constructs, like empathy, there are no standardized instruments designed to capture medical student idealism.

A more general limitation to this study, and to any study of idealism loss in medical students at this point in time, is the fact that it is unclear whether a loss of idealism is not simply a natural maturation process, and in some ways desirable. For example, a decline in idealism may reflect a concurrent increase in necessary or useful traits such as pragmatism or resiliency. Sethia and others have argued for the preservation of idealism in the face of pragmatism [10], but certainly resiliency is a beneficial characteristic for medical practice. Additionally, the loss of idealism may act as a filter for those who would ultimately not thrive in a resource-poor or otherwise challenging environment. However, in the face of perpetual shortages in primary and underserved care workforces, the prospect of losing potential physicians who are interested in working in these environments to the attrition of ideals is unfortunate, at best.

## Conclusions

Although this study did not investigate the reasons behind this downward shift of idealism, it does suggest the need for earlier intervention to maintain the idealism of future medical professionals. Previous studies investigating the maintenance of idealism in medical students and residents have linked the decline of idealism to an increased disinterest in providing care to underserved populations [1,10]. The current context of health care reform under the Patient Protection and Affordable Care Act (ACA) in the United States has a marked focus on increasing access to care for underserved and marginalized groups. The coverage expansions of the ACA in the U.S. will accelerate demand for an already under-supplied physician workforce in coming years [28,29]. Addressing the decline in idealism among medical students may be one avenue through which medical schools can work to increase the number of trained physicians who chose to practice in primary care settings and provide care to underserved populations.

Identifying the point at which the downward shift in idealism occurs, coupled with an understanding of the agents for this change, is an essential step in the development of strategies for the preservation of idealism in medical students. Medical students are confronted with different challenges and experiences in each stage of the medical curriculum; pinpointing the stages during which idealism most noticeably fades can provide a target for the implementation of potential interventions. For example, results from a study utilizing self-reported data indicate that activities such as international electives, or electives held in community settings, can have a positive impact on student attitudes as they relate to idealism [1], and appropriately timing such coursework in the medical curriculum can yield the optimal benefit to student idealism. Additional work towards the development of appropriate interventions aimed at preserving idealistic intentions is warranted, and identifying time points in a traditional medical curriculum where idealism suffers is a key step in facilitating such work.

The findings of our research support and strengthen the conclusion that the decline in medical student idealism begins as early as the second year of medical school. Although our observations do not follow a narrowly defined student cohort, they do provide several measures of student idealism across all years of medical education, and thus paint a broad picture of the change in medical student attitudes toward a career in medicine. Additionally, the findings of this study provide insight into factors that may influence these changing attitudes as medical students progress through their curriculum. Future investigations into the mechanisms behind the loss of idealism are warranted if we are to train an adequate number of physicians exemplifying a focus on care for the underserved.

## Abbreviations

MS1: First year medical student; MS2: Second year medical student; MS3: Third year medical student; MS4: Fourth year medical student; T1: Beginning of 2010/2011 academic year; T2: End of 2010/2011 academic year; AY2010: 2010/2011 academic year; AY2012: 2012/2013 academic year; LCV: Linear composite variable; PCA: Principal component analysis; RUCA: Rural–urban commuting area.

## Competing interests

The authors declare that they have no competing interests.

## Authors’ contributions

EMM assisted in all analyses of the data presented here and contributed to the narrative report. CR co-designed and administered the survey, managed the data as they were collected, and contributed to the narrative report. CPM designed the study, led the analysis, and contributed to the narrative report. All authors read and approved the final product.

## Pre-publication history

The pre-publication history for this paper can be accessed here:

http://www.biomedcentral.com/1472-6920/14/58/prepub

## References

[B1] Smith JK Weaver DB Capturing Medical Students’ Idealism Ann Fam Med 2006 4 S32 37 10.1370/afm.543 17003160 PMC1578670

[B2] Woloschuk W Harasym PH Temple W Attitude change during medical school: a cohort study Med Educ 2004 38 522 534 10.1046/j.1365-2929.2004.01820.x 15107086

[B3] Pagnin D De Queiroz V De Oliveira Filho MA Gonzalez NV Salgado AE Cordeiro e Oliveira B Lodi CS Melo RM Burnout and career choice motivation in medical students Med Teach 2013 35 388 394 10.3109/0142159X.2013.769673 23458255

[B4] Chen DC Kirshenbaum DS Yan J Kirshenbaum E Aseltine RH Characterizing changes in student empathy throughout medical school Med Teach 2012 34 305 311 10.3109/0142159X.2012.644600 22455699

[B5] Chen D Lew R Hershman W Orlander J A cross-sectional measurement of medical student empathy J Gen Intern Med 2007 22 1434 1438 10.1007/s11606-007-0298-x 17653807 PMC2305857

[B6] Griffith CH 3rd Wilson JF The loss of student idealism in the 3rd-year clinical clerkships Eval Health Prof 2001 24 61 71 10.1177/01632780122034795 11233586

[B7] Hojat M Vergare MJ Maxwell K Brainard G Herrine SK Isenberg GA Veloski J Gonnella JS The devil is in the third year: a longitudinal study of erosion of empathy in medical school Acad Med 2009 84 1182 1191 10.1097/ACM.0b013e3181b17e55 19707055

[B8] Crandall SJ Reboussin BA Michielutte R Anthony JE Naughton MJ Medical students’ attitudes toward underserved patients: a longitudinal comparison of problem-based and traditional medical curricula Adv Health Sci Educ Theory Pract 2007 12 71 86 10.1007/s10459-005-2297-1 17041814

[B9] Morley CP Roseamelia C Smith JA Villarreal AL Decline of medical student idealism in the first and second year of medical school: a survey of pre-clinical medical students at one institution Med Educ Online 2013 18 21194 23968751 10.3402/meo.v18i0.21194 PMC3750194

[B10] Sethia B In praise of idealism in healthcare J R Soc Med 2013 106 344 5 10.1177/0141076813499277 23995821 PMC3758676

[B11] Warwick MT Idealism includes compassion and commitment to patients J R Soc Med 2013 106 474 10.1177/0141076813509148 24284995 PMC3842858

[B12] Abbasi K Idealism, the lost spirit of medicine J R Soc Med 2013 106 343 10.1177/0141076813501787 23995820 PMC3758682

[B13] Enoch L Chibnall JT Schindler DL Slavin SJ Association of medical student burnout with residency specialty choice Med Educ 2013 47 173 181 10.1111/medu.12083 23323656

[B14] Newton BW Barber L Clardy J Cleveland E O’Sullivan P Is there hardening of the heart during medical school? Acad Med 2008 83 244 249 10.1097/ACM.0b013e3181637837 18316868

[B15] Newton BW Walking a fine line: is it possible to remain an empathic physician and have a hardened heart? Front Hum Neurosci 2013 7 233 23781181 10.3389/fnhum.2013.00233 PMC3678078

[B16] Winseman J Malik A Morison J Balkoski V Students’ views on factors affecting empathy in medical education Acad Psychiatry 2009 33 484 91 10.1176/appi.ap.33.6.484 19933894

[B17] Phillips SP Clarke M More than an education: the hidden curriculum, professional attitudes and career choice Med Educ 2012 46 887 93 10.1111/j.1365-2923.2012.04316.x 22891909

[B18] Phillips SP Blinded by belonging: revealing the hidden curriculum Med Educ 2013 47 124 5 10.1111/medu.12103 23323650

[B19] Burks DJ Kobus AM The legacy of altruism in health care: the promotion of empathy, prosociality and humanism Med Educ 2012 46 317 25 10.1111/j.1365-2923.2011.04159.x 22324531

[B20] Hafler JP Ownby AR Thompson BM Fasser CE Grigsby K Haidet P Kahn MJ Hafferty FW Decoding the Learning Environment of Medical Education: A Hidden Curriculum Perspective for Faculty Development Acad Med 2011 86 440 444 10.1097/ACM.0b013e31820df8e2 21346498

[B21] Woloschuk W Wright B McLaughlin K Debiasing the hidden curriculum: academic equality among medical specialties Can Fam Physician 2011 57 e26 30 21252122 PMC3024184

[B22] Holmes D Tumiel-Berhalter LM Zayas LE Watkins R “Bashing” of medical specialties: students’ experiences and recommendations Fam Med 2008 40 400 406 18773777

[B23] Griffith CH Wilson JF The loss of idealism throughout internship Eval Health Prof 2003 26 415 426 10.1177/0163278703258107 14631612

[B24] Youngclaus JA Koehler PA Kotlikoff LJ Wiecha JM Can medical students afford to choose primary care? An economic analysis of physician education debt repayment Acad Med 2013 88 16 25 10.1097/ACM.0b013e318277a7df 23165279

[B25] Phillips JP We must make the cost of medical education reasonable for everyone Acad Med 2013 88 1404 24064611 10.1097/ACM.0b013e3182a2b013

[B26] Phillips JP Weismantel DP Gold KJ Schwenk TL Medical student debt and primary care specialty intentions Fam Med 2010 42 616 22 20927669

[B27] Phillips J Weismantel D Gold K Schwenk T How do medical students view the work life of primary care and specialty physicians? Fam Med 2012 44 7 13 22241335 PMC4120645

[B28] Carrier ER Yee T Stark LB Matching Supply to Demand: Addressing the U.S. Primary Care Workforce Shortage 2011 NIHCR Policy Anal No. 7. http://www.nihcr.org/PCP_Workforce

[B29] Allen SM Ballweg RA Cosgrove EM Engle KA Robinson LR Rosenblatt RA Skillman SM Wenrich MD Challenges and opportunities in building a sustainable rural primary care workforce in alignment with the Affordable Care Act: the WWAMI program as a case study Acad Med 2013 88 1862 9 10.1097/ACM.0000000000000008 24128621

